# The Virus-Host Interplay: Biogenesis of +RNA Replication Complexes

**DOI:** 10.3390/v7082825

**Published:** 2015-08-06

**Authors:** Colleen R. Reid, Adriana M. Airo, Tom C. Hobman

**Affiliations:** 1 Department of Medical Microbiology and Immunology, University of Alberta, Edmonton, AB T6G 2E1, Canada; E-Mails: crreid1@ualberta.ca (C.R.R.); airo@ualberta.ca (A.M.A.); 2 Department of Cell Biology, University of Alberta, Edmonton, AB T6G 2H7, Canada

**Keywords:** +RNA viruses, replication complexes, host factors, membranes

## Abstract

Positive-strand RNA (+RNA) viruses are an important group of human and animal pathogens that have significant global health and economic impacts. Notable members include West Nile virus, Dengue virus, Chikungunya, Severe acute respiratory syndrome (SARS) Coronavirus and enteroviruses of the *Picornaviridae* family.Unfortunately, prophylactic and therapeutic treatments against these pathogens are limited. +RNA viruses have limited coding capacity and thus rely extensively on host factors for successful infection and propagation. A common feature among these viruses is their ability to dramatically modify cellular membranes to serve as platforms for genome replication and assembly of new virions. These viral replication complexes (VRCs) serve two main functions: To increase replication efficiency by concentrating critical factors and to protect the viral genome from host anti-viral systems. This review summarizes current knowledge of critical host factors recruited to or demonstrated to be involved in the biogenesis and stabilization of +RNA virus VRCs.

## 1. Introduction

Positive-sense RNA (+RNA) viruses including the Flaviviruses, enteroviruses of the *Picornaviridae* family, Alphaviruses, and Coronaviruses all dramatically modify cellular membranes to serve as platforms for replication and assembly of new virions. The biogenesis of these replication compartments is a complex interplay of interactions between virus and host proteins. Although considerable progress has been made in identifying host proteins that interact with virus-encoded proteins, much remains to be learned regarding the significance of these interactions. Despite morphological differences in the replication complexes formed by members of each viral family, these viruses have evolved to use common cellular pathways to complete biogenesis. Some of the shared pathways highlighted in this review include lipid metabolism, autophagy, signal transduction and proteins involved in intracellular trafficking (Table 1). Remarkably, even within the higher order of shared pathways, differences within members of specific families (such as *Flaviviridae*) exist, highlighting that the assembly and function of viral replication complexes (VRCs) varies considerably. As such, this review focuses on a broad view of host factors in which there is significant functional evidence linking them to VRCs in effort to highlight commonalities or differences and further advance the understanding of virus-host interactions.

## 2. *Flaviviridae*

The *Flaviviridae* family includes many significant global pathogens including Hepatitis C virus (HCV), West Nile virus (WNV), and Dengue virus (DENV). This family is comprised of four genera, with the human pathogens belonging to the genera *Flavivirus* and *Hepacivirus*. The *Hepacivirus* genus contains HCV, a prominent blood-borne human pathogen that causes chronic hepatitis and is estimated to have infected 170 million people worldwide. The *Flavivirus* genus includes DENV, WNV, Yellow Fever virus (YFV) and other viruses causing either haemorrhagic or encephalitic disease. Except for YFV and Japanese Encephalitis virus (JEV), vaccines for use in humans are not available against members of this family. Current treatment options are very limited and supportive care is often the only option. Arthropod vectors, mainly mosquitos and ticks are used by most flaviviruses to infect their hosts.

In general, virions are enveloped and contain a single copy of viral genomic RNA (~11 kilobase (kb)) encoding a single polyprotein that is cleaved by viral and host proteases into three structural and seven non-structural proteins [1]. After binding to cell surface receptors, the virions enter cells through endocytic pathways. Within the acidic environment of endosomes, the virions fuse with endosomal membrane resulting in release of the nucleocapsid into the cytoplasm. After the nucleocapsid disassembles, the viral RNA is translated into a polyprotein, which is then processed into individual viral proteins. VRCs form soon after and serve as platforms for RNA replication. Assembly of nascent virions occurs in close proximity to VRCs on the endoplasmic reticulum (ER). After budding into the ER, virions traverse the secretory pathway before release from the cell.

### 2.1.Genus Hepacivirus

The biogenesis of the HCV VRCs and the stabilization of these structures have been extensively studied [2,3]. Electron microscopic analysis of infected cells revealed that HCV replicates on altered ER membranes that are closely associated with lipid droplets; termed the “membranous web” [4].The membrane-associated non-structural protein 4B (NS4B) plays a key role in the formation of this network [5], which consists of double membrane vesicles (DMVs) protruding out of ER. Of note, the DMVs are similar to ER-associated structures induced by members of *Picornaviridae* and *Coronaviridae* [6]. A plethora of host factors involved in lipid metabolism, intracellular signalling, protein folding, and vesicular trafficking are known to be important for HCV VRC activity. Due to the availability of extensive literature on the subject, they will not be discussed here. Instead, we refer readers to the following recent reviews [3,7,8].

### 2.2. Biogenesis of the Flavivirus Replication Complex

**Figure 1:**
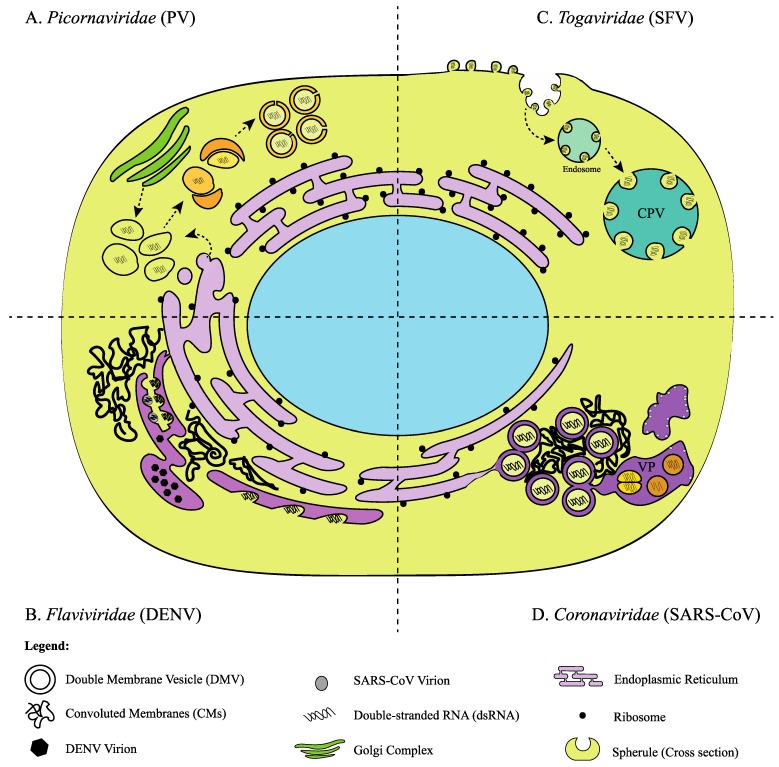
Biogenesis of Viral Replication Complexes (VRCs): Representative diagram of the structure and biogenesis of the VRCs for each family based on electron microscopy from the following references: Poliovirus (PV) [23] Semliki Forest virus (SFV) [24], Dengue virus (DENV) [15], Severe acute respiratory syndrome coronavirus (SARS-CoV) [25]. Diagram not to scale. (**A**) Formation of the PV VRC: Early in infection single membrane vesicles that contain dsRNA are derived from the ER and Golgi components. Progression of infection results in vesicles wrapping around each other inducing the formation of DMVs; (**B**) Formation of the DENV VRC: Spherule structures containing dsRNA form on modified rough-ER membranes surrounded by convoluted membranes. Assembly sites form on opposing cisternae where newly formed virions are stored; (**C**) Formation of SFV VRC: Spherules that contain dsRNA form at the plasma membrane. Internalization of these structures follows the endo-lysosomal pathway resulting in formation of cytopathic vacuoles (CPV); (**D**) Proposed formation of the SARS-CoV VRC: DMVs containing dsRNA are formed and their outer membranes are continuous with the ER. Convoluted membranes surround the DMVs. Formation of vesicle packets (VPs) is thought to result from DMV fusion. Newly formed virions are associated with these structures.

Of the studies investigating the membrane alterations induced by members of this genus, most have focused on DENV and WNV. Infection of mammalian cells with either the Australian attenuated strain WNV_KUN_, or the highly pathogenic WNV_NY99_ strain results in similar phenotypic disruptions of cellular membranes [9,10]. Early studies of cells infected with WNV or DENV revealed dramatic changes in cellular membranes and the formation of single membrane vesicular packets (VPs) and convoluted membranes (CM), which are in close association with smooth membranes and the rough-ER [9,11]. Paracrystalline arrays (PC) were also described in WNV_KUN_-infected cells [9]. Infection of cells derived from the viral vector (mosquito) with DENV or WNV also led to dramatic alterations of membranes resulting in spherules associated with ER membranes [12,13]. These virus-induced structures are thought to segregate viral replication from protein translation [14]. VPs are the sites of viral replication as evidenced by the fact that they contain double-stranded RNA (dsRNA), a replication intermediate, and the viral RNA-dependent RNA polymerase, NS5 [9,11,15,16,17]. Two other virus-encoded non-structural proteins NS1 and NS3 also associate with these elements. VPs in DENV- and WNV-infected cells are ~85 nm in diameter indicating the conserved nature of these structures. CMs and PCs in WNV_KUN_-infected cells are enriched for NS3/2b, the viral protease and do not contain dsRNA [9,15]. This suggests that CM/PCs may be the sites of viral translation and/or proteolytic processing of the viral proteins. The origins of these membranes vary between viruses. DENV-induced VP membranes contain the ER resident proteins protein disulphide isomerase and calnexin [15], whereas in cells infected with WNV_KUN_, VPs that are positive for dsRNA, contain the *trans*-Golgi network protein, galactosyltransferase, possibly indicating that these structures are derived from Golgi membranes [18]. Moreover, the ER-Golgi intermediate compartment marker ERGIC53, is associated with CMs and PCs. WNV_NY99_ is similar to DENV in that the VRCs colocalize with protein disulphide isomerase, suggesting these structures are ER-derived [10]. Electron tomography was utilized to further characterize the VRCs of DENV [15] (Figure 1B), WNV_KUN_ [9,19], WNV_NY99_ [20], and Tick-borne encephalitis (TBEV)-like virus Langat virus [21]. These “vesicles” in fact appear to be invaginations of the ER membrane with small neck-like openings (~11.2 nm for DENV) that may facilitate trafficking of molecules into and out of these replication sites. In some cases, there were connections between these vesicles within the modified-ER membrane. DENV VPs closely associated with budding sites appear as electron dense invaginations (~60 nm) and can be seen on opposing cisternae [15]. Despite there being good structural information on DENV and WNV VRCs, the exact mechanism by which these membranous organelles form, remains unclear. However, the ER localized non-structural viral proteins, WNV_NY99_ NS4B [20] and DENV-2 NS4A [22] are thought play a role in the initial membrane curvature.

Following this step, other viral and host factors are likely required for the biogenesis and stabilization of the flavivirus VRC. To date, a large number of host proteins involved in flavivirus replication have been identified by proteomic and transcriptomic studies of infected cells [26,27,28], mapping the host cell interactome of viral proteins [29,30,31] and through systematic RNA interference (RNAi) screens [32,33]. Perhaps not surprisingly, common host pathways that affect flavivirus replication include those involved in lipid metabolism, signal transduction, and cell structure. While many host factors that are thought to play a role in virus replication have been identified for WNV and DENV, the corresponding functional and validation studies are comparatively limited. As such, in this review, we have focused mainly on host factors in which there are significant functional data linking them to VRCs.

### 2.3. Potential Role for Autophagy in Flavivirus VRC Biogenesis

Autophagy is a homeostatic process involving the formation of double membrane vesicles from the ER that fuse with lysosomes and degrade cellular material. Recently, it has been linked to VRC biogenesis for multiple viruses, including the *Picornaviridae* and *Coronaviridae* family (covered later in this review). The requirement for autophagy in flavivirus VRCs varies significantly. WNV propagation for example, is not affected by induction or repression of autophagy [34], nor is autophagy required for biogenesis of VRCs from the ER [35]. This is in contrast with DENV and JEV, which both exploit autophagy for virus propagation [36,37]. It has been proposed that DENV uses autophagy-induction to aid in release of fatty acids from lipid droplets, increase β-oxidation and ATP production [38]. Little is known about the role of autophagy in VRC biogenesis or replication of other members of the *Flavivirus* genus.

### 2.4. Membrane Remodelling and Lipid Metabolism

Biogenesis of VRCs requires massive expansion of ER-associated membranes and alteration of their lipid compositions. HCV and members of the *Picornaviridae* family are known to recruit phosphatidyl-inositol kinases (PI4Ks) to their VRC networks for the conversion of Phosphatidylinositol (PI) to Phosphatidylinositol 4-phosphate (PI4P) lipids, a process that is essential for viral replication [39,40]. PI4P lipids may serve to recruit host proteins and/or lipid components to these organelles. Interestingly, neither WNV nor DENV seem to require PI4P lipids [41,42] indicating that assembly and function of VRCs varies considerably among the *Flaviviridae* family. However, DENV infection does alter the membrane lipid composition of human cells and two host cell enzymes involved in fatty acid metabolism, fatty acid synthase (FAS) and Acetyl-CoA carboxylase 1 (ACACA) are both important for replication [42]. FAS is recruited to the VRC through interaction with the viral protease NS3, where it upregulates the formation of fatty acids from acetyl-CoA. As the length of fatty acid chains can affect membrane curvature, this process may be important for the formation of the VRC [43].

Newly synthesized fatty acids are incorporated into DENV VRCs and pharmacological inhibition of FAS by cerulenin or C75 negatively affects DENV replication in mammalian [42] as well as mosquito cells [44]. In one current model, the viral protein NS4A initially induces membrane curvature followed by the recruitment of FAS by NS2B/NS3 to the VRC resulting in local production of fatty acids and the expansion of the ER membrane in a more fluid state [42]. Similar to DENV, WNV requires FAS activity for replication [41]. WNV_NY99_ infection also increases the intracellular concentration of sphingolipids and glycerophospholipids, a process that affects the make up of virion envelopes [45]. Virus assembly and release is also dependent on lipid biosynthesis, particularly ceramide. The role of glycerophospholipids such as phosphatidylcholine (PtdCho) in flavivirus replication is less clear, but there is evidence that PtdCho is part of the VRCs and is incorporated into the lipid bilayers of nascent virions [45].

The level of cholesterol also modulates the curvature and plasticity of membranes [46]; a process that is controlled by ER-localized transcription factor sensors, sterol-regulatory element binding proteins (SREBPs). When cholesterol levels are low, SREBP is released from the ER and enters the nucleus where it activates transcription of the genes for FAS, 3-hydroxy-methyglutaryl-CoA reductase (HMGCR), and/or low-density lipoprotein receptor (LDLR). HMGCR catalyzes the rate-limiting step in synthesis of mevalonate, a precursor for cholesterol biosynthesis, whereas LDLR is a cell surface receptor that binds and internalizes cholesterol-containing complexes [47,48]. It is well documented that flaviviruses modulate cholesterol levels in infected cells. During WNV_KUN_ infection, total cholesterol levels rise and this is correlated with upregulation and association of HMGCR with virus-induced membrane structures [49]. This may indicate HMGCR aids VRC formation by producing cholesterol at these sites.

Interestingly, elevated cholesterol (total) levels were not observed in DENV-infected cells [50], even though LDLR transcripts, an indicator of elevated ER cholesterol, were increased. However, inhibition of HMG-CoA reduced replication of DENV replicons indicating cholesterol is important for replication. Another host factor involved in cholesterol biosynthesis, mevalonate diphosphate decarboxylase (MVD), was shown to be important for DENV replication [50]. Thus cholesterol seems to be a key lipid component of the flavivirus VRC and while total cellular cholesterol may not increase in all flavivirus infected cells, this membrane is targeted to the ER membrane where VRCs are produced. Future studies with JEV, YFV, TBEV, and St. Louis Encephalitis Virus (SLEV), are needed to determine how they may alter membrane composition in favour of VRC biogenesis.

### 2.5. Stabilization and Scaffolding Proteins at the Flavivirus VRC

Microfilaments, microtubules and intermediate filaments are cytoskeletal components essential for cell shape and motility as well as a myriad of other functions including intracellular trafficking, cell division and cell signalling. Identification of host proteins involved in actin polymerization and vesicular trafficking were shown to be important for DENV and WNV replication [32,42], however, comparatively little is known about how this affects VRC formation or function. Conceivably, changes to the cellular structural framework could aid in biogenesis and/or stabilization of newly formed VRCs. Reorganization of the intermediate filament component vimentin occurs after phosphorylation by calcium/calmodulin-dependent protein kinase II; an event that is necessary for productive DENV replication [51]. Moreover, knockdown of vimentin alters the distribution of the VRCs in host cells, indicating that this protein may function in scaffolding/stabilization of these structures. NS4A interactions with vimentin may be the link between intermediate filaments and VRCs [51]. Finally, Stathmin 1 (STMN1), a microtubule destabilizing protein, is another host factor linked to biogenesis of VRCs [52]. DENV infection upregulates STMN1 by reducing levels of miR-223, a microRNA that normally targets the mRNA for STMN1.

## 3. *Picornaviridae*

The *Picornaviridae* family contains many important human and animal pathogens. Prior to the development of a vaccine, poliovirus (PV) crippled hundreds of thousands of people per year, primarily children. The World Health Organization global PV eradication program started in 1988 has not yet been successful in fully eradicating the virus. Other prominent members of this family include Coxsackie virus (CV), human rhinoviruses (HRV), and the causative agent of hand-foot-and-mouth disease Enterovirus 71 (EV71). Unlike PV, effective vaccines against these pathogens have yet to be developed. Infection by CV, HRV, and EV71 cause a variety of illnesses in humans from self-limiting colds to more serious presentations of encephalitis, myocarditis, and paralysis. Children, elderly, and the immuno-compromised individuals are at highest risk for severe disease.

The majority of research has focused on members of the *Enterovirus* genus including PV, CV, and HRV, and as such our focus will be on host factors linked to formation and stabilization of their VRCs. As with all +RNA viruses, enteroviruses extensively rearrange cellular membranes to facilitate virus replication and assembly. Following entry of the virion, the 5′capped genomic RNA (~7.5 kb) is unpackaged after which translation is initiated from an internal ribosome sequence. The genomic RNA encodes a single large polyprotein that is proteolytically processed into four structural proteins that form the virion, and seven non-structural proteins that function in replication and subverting the host-cellular immune system.

### 3.1. Biogenesis of the Enterovirus VRC

Early electron microscopy studies of PV-infected cells by Dales and Palade revealed drastic remodelling of the cell cytoplasm [53]. At 5 hours post-infection (hpi), membrane-enclosed bodies were observed in the perinuclear zone. At the peak of viral translation (2.5 hpi), nascent VRCs were not observed but rather, formed later during RNA replication [54]. Isolation of PV VRCs revealed that in addition to dsRNA, a replication intermediate, proteins encoded by the P2 genomic region, specifically 2C containing non-structural proteins, were bound to these membranes [55,56]. More recently, electron tomographic studies were used to examine the biogenesis of these structures in more detail [23]. Early in infection (~2 hpi), 100–200 nm single membrane tubular structures that are involved in RNA synthesis form followed by clustering and bending of these structures at 4 hpi. Later, DMVs, which can be larger (100–300 nm), form through a membrane wrapping process (Figure 1A). These structures evolve from *cis*-Golgi membrane and arise from positive membrane curvature or budding [23]. Despite a tremendous amount of experimental investigation, the origins of the enteroviral VRC remain controversial [57].

### 3.2. Membrane Remodelling during Enteroviral Infection

Earlier studies suggested that PV VRCs are derived from multiple membrane sources, including lysosome, ER, and Golgi, but do not fully resemble their parent membrane sources [58,59]. Multiple hypotheses exist for how these structures arise during PV infection, including through autophagy [58]. Expression of the PV proteins 2BC and 3A results in the formation of DMVs [59] that colocalize with lysosomal-associated membrane protein 1 (LAMP-1) and LC3-phosphatidylethanolamine conjugate (LC3-II), indicative of autophagic vesicles, early in PV infection [60]. Data consistent with the PV studies were observed with the related enteroviruses HRV-2 and HRV-14 and inhibition of autophagy decreased the amount of intracellular and extracellular virus produced [60,61,62,63]. Of note, CVB3 infection induces autophagy in a mouse model *in vivo*, indicated by increased LC3-II [61]. Despite this evidence, the role of autophagy remains controversial. Recently, it was shown that PV vesicles that stained for dsRNA did not colocalize with autophagic marker LC3 early (3 hpi) in infection [64]. However, at 5 hpi LC3 was detected by immuno-electron microscopy in association with dsRNA. In light of the seemingly discrepant data, it has been postulated that autophagy is important for late steps in infection (3 hpi) [64].

COPII-coated vesicles, which are involved in transport of cargo from the ER, have been linked to biogenesis of enterovirus VRCs. During early infection, enteroviruses disrupt anterograde transport and reroute these vesicles to sites of viral replication. PV infection or expression of protein 3A alone has been shown to block ER-Golgi protein transport [65,66]. Moreover, the movement of VRCs is dependent on microtubules leading from the ER to the microtubule organizing center in the Golgi region of the cell [67]. PV proteins 2B and its precursor 2BC colocalize with the COPII component Sec31 [68] and PV infection enhances COPII vesicle budding [69]. However, the effect is transient and is not observed late in infection. In contrast, HRV-1A and -16 have been reported to cause fragmentation of the Golgi without blocking protein secretion [70,71]. Furthermore, recent evidence citing lack of colocalization between dsRNA and the COPII component Sec31 has been interpreted to mean that PV VRC formation is not dependent on COPII [64]. One potential mechanism to account for this discrepancy is that COPII aids in the formation of an intermediary compartment from which nascent VRCs bud. However, attenuation of COPII vesicle formation did not interfere with PV infection suggesting that this budding mechanism is not absolutely required [72]. Clearly, more research is required to fully understand the role of COPII in picornavirus VRC biogenesis and function.

PV infection is sensitive to brefeldin A (BFA), a drug that inhibits activation of ADP-ribosylating factor GTPases (Arfs), which are necessary for formation of COPI vesicles [73,74]. Arfs cycle between a GDP (inactive) and a GTP (active) bound state mediated by guanine-nucleotide exchange factors (GEFs). When bound to GTP, Arfs remodel intracellular membranes to promote COPI dependent budding [75]. COPI mediates budding of vesicles from the Golgi that retrograde traffic to the ER and was identified as a host factor that is required for replication of *Drosophila* C virus, a picorna-like insect virus [72]. Reducing expression of α-COP, a COPI component, was later found to reduce PV infection [72]. Expression of PV 3A or 3CD promotes the association of Arf3 and Arf5 with membranes where viral RNA replication occurs, and this association can be blocked by BFA [76]. Two other GEFs, BIG1 and BIG2 are recruited to VRCs by expression of PV 3CD which then leads to the activation of Arf [77]. This indicates that Arf activation may induce vesicle formation and VRC biogenesis (reviewed in [78]). GBF1, yet another GEF, is also a target of BFA and is the main activator of Arf during PV infection [79]. PV 3A binds GBF1 and recruits it to virus-induced vesicles [77]. While VRCs can still form in the presence of BFA, they are unable to recruit Arf1. This may result in formation of defective VRCs or mislocalization of their contents thereby reducing viral replication and assembly [79]. Although BFA targets BIG1, BIG2, and GBF1, only GBF1 is required for CVB3 replication [80].

More recent studies examined the localization of Arf1 and GBF1 throughout CVB3 and PV infections [39]. Arf and GBF1 colocalize with viral RNA and the viral RNA polymerase, indicating their relocalization to VRCs during infection. Moreover, Arf1 knockdown negatively impacts virus replication [39]. However, since Arf1 interacts strongly with GBF1 and is found in COPI vesicles, it cannot be ruled out that recruitment of Arf1 to the VRC is a consequence of GBF1 recruitment. β-COP, a COPI component, does not colocalize with dsRNA during PV infection and thus, the COPI coat itself may not be involved budding and biogenesis of VRCs [64]. Recruitment of Arf1/GBF1 to VRCs appears to differ among enteroviruses. Expression of CVB3 3A induces the recruitment of GBF1 to membranes, whereas the homologous proteins of HRV-2 or -14 do not [81,82]. These viral proteins also function in recruitment of Phosphatidylinositol-4-OH kinase type III beta (PI4KIIIβ) to VRCs and this is discussed in further detail below.

### 3.3. Lipid Metabolism

Biogenesis of picornavirus VRCs also requires synthesis and trafficking of specific lipids to membrane organelles. Unlike enveloped viruses such as flaviviruses and togaviruses (also covered in this review) whose replication compartments are formed by invagination into membranes, picornaviruses induce protrusion of cellular membranes to form convoluted tubular-like structures [23,83]. Alterations in the lipid composition of these membranes are needed to allow appropriate curvature and the expansion of membranes that eventually form the VRCs. Early evidence of altered lipid metabolism came from the observation that PV increases PtdCho levels in the cells by upregulating the rate-limiting enzyme phosphocholine cytidylyltransferase [84]. PtdCho is a main component of lipid bilayers at the ER and Golgi network and increased levels of this phospholipid would enable proliferation of membranes (reviewed in [85]). This may indicate that formation of VRCs involves *de novo* lipid synthesis. The role of fatty acids was first reported when the addition of cerulenin, an inhibitor of the enzyme FAS, resulted in a block of PV replication but did not affect viral RNA translation or proteolytic processing [86]. Blocking fatty acid synthesis by inhibiting FAS also reduced proliferation of VRC membranes. Later, the same group reported that specific fatty acids are important for PV replication as evidenced by the observation that incorporation of oleic acid into cellular membranes made them incapable of supporting PV replication [87]. More recently the role of FAS for CVB3 replication has also been demonstrated. FAS upregulation was first observed during a proteomic screen of CVB3 infected cells [88]. FAS protein production is upregulated as early as one hour post CVB3 infection and does not require viral replication, suggesting that FAS gene transcription and translation may be upregulated following signalling cascades induced by CVB3 virions binding [89]. Components of the fatty acid biosynthesis pathway including SREBP and the protein product of the gene it directly regulates, *CG3523* encoding FAS are also required for picorna-like virus *Drosophila C* virus replication, suggesting that FAS may be a common host factor exploited by viruses to alter membrane lipid metabolism [72]. In addition to altering metabolism in cells, picornaviruses may increase uptake of lipids from the extracellular environment. PV infection for example, enhances import of long-chain fatty acids into cells and the viral protein 2A is involved in the initiation of this process [90]. Normally, fatty acids are trafficked and stored in lipid droplets, but in infected cells they colocalize with VRCs. Moreover, activity of long chain acyl-coenzyme A (Acyl-CoA) synthetase Acsl3, involved in the synthesis of PtdCho, was upregulated at 2 hpi, thus further supporting the notion that VRCs are formed from newly synthesized lipids.

Because Arf1 and GBF1 are recruited to VRC membranes, it was thought that Arf1 effectors might also be important for enterovirus replication. Lipid modifying enzymes including (PI4Ks) are downstream effectors of Arf. PI4KIIIβ is normally associated with the Golgi and is involved in the production of (PI4P) [91]. The PI4KIIIβ inhibitor enviroxime exhibits potent antiviral activity against enteroviruses *in vitro*, however in clinical trials, its efficacy was limited [92,93]. Other PI4KIIIβ inhibitors, including GW5074 and BF738735, also efficiently inhibit enteroviral replication *in vitro* and *in vivo* in mice, however in some mice strains inhibition of PI4KIIIβ resulted in harmful side effects thereby limiting the likely therapeutic benefit of this strategy [92,94,95]. PI4KIIIβ colocalizes with Arf1 at replication complexes during CVB3 infection, while other Arf1 effectors, such as COPI components, are lost from these sites [39]. Recruitment of PI4KIIIβ to Arf1-positive membranes can be stimulated by expression of CVB3 3A alone. During infection by PV or CVB3, PI4P levels increase 5-fold and pharmacological inhibition of PI4KIIIβ activity by PIK93, which inhibits PI4P production, also reduces viral replication. Furthermore, PI4KIIIβ interacts with CVB3 3D^pol^, which strongly interacts with PI4P-containing membranes and thus their production may facilitate the organization and/or association of viral proteins in the VRC [39]. This kinase also interacts with PV 3A and Acyl-CoA binding domain containing 3 (ACBD3) protein [96]. Recruitment of PI4KIIIβ to the VRC seems to be conserved among enteroviruses but the interactions between this kinase and viral as well as other host proteins remain to be fully elucidated.

The production of PI4P lipids, by PI4KIIIβ at replication sites may also be important for recruitment of cholesterol. Both PI4P and cholesterol are enriched at VRCs of CVB3, PV, and HRV-2 [94]. Recently, it was suggested that cholesterol is shuttled from the endosome to the VRC where it colocalizes with CVB3 3A [97] suggesting that the virus re-routes pre-existing pools of cholesterol to VRCs. Moreover, it has also been demonstrated that uptake of extracellular cholesterol by clathrin-mediated endocytosis (CME) is essential for PV and CVB3 replication [98]. After uptake, cholesterol is targeted to recycling endosomes, which then fuse with existing VRCs. Depletion of CME components results in a trafficking of cholesterol from the plasma membrane to lipid droplets, reducing VRC formation [98]. Incorporation of cholesterol into VRCs, which imparts rigidity to membranes, may be important for their curvature and stabilization.

## 4. *Coronaviridae*

Members of the four genera in the family *Coronaviridae* have enveloped virions that contain very large +RNA, capped and polyadenylated RNA genomes of 26–32 kb. These viruses infect a wide range of mammals and birds causing upper respiratory, gastrointestinal, hepatic, or central nervous system diseases [99]. Members belonging to the genus *Betacoronavirus* include the important human pathogens Severe acute respiratory syndrome coronavirus (SARS-CoV) and Middle East respiratory syndrome coronavirus (MERS-CoV). Prior to the SARS outbreak in 2003, the majority of coronavirus (CoV) research focused on mouse hepatitis virus (MHV) as a model.

Following virion entry into host cells, the +RNA genome is released into the cytoplasm. Coronaviruses employ a rather complex program of gene expression. The *ORF1* encodes the replicase required for transcription of the full-length (genomic) minus-strand template and subgenomic (discontinuous transcription) minus-strand synthesis. Synthesis and processing of the genome results in production of up to 16 nonstructural proteins, of which the predicted multi-spanning membrane proteins nsp3 [100], nsp4 [101] and nsp6 [102] are believed to be involved in biogenesis and stability of the coronavirus replication/transcription complex (RTC). The study of host factors required for biogenesis and stabilization of the coronavirus RTC is a growing field of interest. The involvement of lipid rafts for virus-entry and cell-cell fusion was demonstrated for MHV [103], however, there is a lack of information on how CoVs modulate host lipid composition as previously shown for many +RNA viruses [104]. A recent kinome screen (using small interfering RNAs) has provided a glimpse of the complexity of pro-viral and anti-viral host factors involved at the SARS CoV-cell interplay, including proteins involved in lipid metabolism [105]. Future studies addressing the interplay between CoVs and lipids and the effects on viral replication would also be of considerable interest.

### 4.1. Coronavirus Replication/Transcription Complex (RTC)

Similar to some of the viruses described above, formation of DMVs is observed during CoV infection in mammalian cells [106]. Early in the SARS-CoV infection process, DMVs, ranging in size from 150 to 300 nm, are distributed throughout the cytoplasm [25]. The ORF1a-encoded multi-spanning transmembrane proteins, nsp3, nsp4 and nsp6 are thought to form the scaffold that facilitates DMV formation and anchors the RTC to intracellular membranes [101,107,108,109]. The RTC is likely formed through a complex network of interactions involving all 16 CoV non-structural proteins [108]. DsRNA can be detected in the interior of DMVs (Figure 1D) [25], suggesting that these structures serve as sites of RNA replication. Indeed this is supported by the observation that RTC activity correlates with the number of DMVs [110]. Electron tomographic analysis revealed that DMVs are not isolated vesicles but rather, an interconnected network of membranes continuous with the rough-ER [25]. Late in infection, DMVs become concentrated in the perinuclear area and CMs of 0.2 to 2 µm in diameter form in close proximity to the DMV clusters [25]. Compared to DMVs, CMs are highly enriched in SARS-CoV nonstructural proteins that include the replicase proteins [25]; leading to the notion that the active replicase complex is localized to the CM and “dead” dsRNA molecules are found in the DMVs, perhaps as a way to evade immune recognition. Electron microscopy studies revealed that similar membrane formations occur in MERS-CoV-infected cells [111]. The outer membranes of DMVs are thought to fuse together and transition into vesicle packets (VPs) late in infection. VPs are large (1–5 µm) membrane structures where virus budding occurs [25,112] (Figure 1D).

Unexpectedly, electron tomographic analyses failed to reveal connections between the interior of DMVs and the cytosol [25]. This suggests that both membranes of CoV DMVs are sealed raising the question of how the import of metabolites and export of viral RNA occurs from these structures. In contrast, small neck-like openings are clearly discernable in DMVs induced by HCV [6]. In this case, it has been speculated that replication takes place in DMVs as long as the connection to the cytosol is maintained. The question remains as to whether CoVs make use of transport molecules as a means to regulate transport of products in and out of DMVs.

**Table 1:** Identified cellular interacting proteins with viral replication complexes of +RNA viruses.

	Lipids and Membrane Remodeling	Cellular Trafficking and Signaling Proteins
***Flaviviridae (Flaviviruses)***	Sphingolipids (WNV_NY99_ [45])	Actin polymerization (WNV [32], DENV [42])
	Glycerophospholipids (WNV_NY99_ [45])	Vimentin (DENV [51])
	FAS (DENV [42], WNV_NY99_ [41])	STMN1 (DENV [52])
	ACACA (DENV [42])	
	Cholesterol (DENV [50], WNV_KUN_ [49])	
***Picornaviridae***	FAS (PV [86,87], CVB3 [88,89])	Arf (CVB3, PV)[39]
	Long chain fatty acids (PV [90])	GBF1 (CVB3, PV)[39]
	PtdCho (PV [84])	
	PI4KIIIβ (CVB3 [39], PV [96])	
	ACBD3 (CVB3, PV) [96]	
	Cholesterol (CVB3, PV) [98]	
***Coronaviridae***		PDI (SARS-CoV [107])
		Sec61α (SARS-CoV [110])
		EDEM1 (MHV [113])
		OS-9 (MHV [113])
***Togaviridae (Alphaviruses)***	Cholesterol and sphingomyelin lipids (SINV [114])	Vimentin (SINV [115])
		PI3K (SFV [116])
		Amphiphysins (SFV, SINV, CHIKV)[117]

Abbreviations: DENV, Dengue Virus; WNV, West Nile Virus; YFV, Yellow Fever Virus; JEV, Japanese Encephalitis Virus; PV, Poliovirus; CVB3, Coxsackievirus B3; HRV-14, Human Rhinovirus 14; EV71, Enterovirus 71; IBV, Infectious Bronchitis virus; SARS-CoV, Severe Acute Respiratory Syndrome coronavirus; MHV, Mouse Hepatitis Virus; SINV, Sindbis Virus; SFV, Semliki Forest Virus; CHIKV, Chikungunya Virus.

### 4.2. Potential Role of Autophagy in DMV Formation

Morphological similarities between CoV DMVs and autophagosomes and the co-localization between specific CoV replicase proteins (nsp8, nsp2, nsp3) with microtubule-associated protein Light chain 3 (LC3), a protein marker for autophagic vacuoles [118], are consistent with autophagy playing a role in DMV formation. Moreover, during MHV infection of murine cells lacking the ATG5 gene which functions in the early stages of autophagosome formation [119], no DMVs formed and virus replication was impaired [120]. Replication was restored by expression of the Atg5 protein further supporting the role of autophagy for formation of DMVs, at least in embryonic stem cells. However, another study involving MHV infection of bone marrow-derived macrophages or embryonic fibroblasts concluded that neither Atg5 nor an intact autophagic pathway is required for viral replication [121]. Other morphological studies also found no evidence for autophagy in DMV formation; specifically a lack of co-localization between the autophagy marker LC3 and the SARS-CoV replication complex [107]. Moreover, MHV replication was unaffected in autophagy-deficient cells although depletion of LC3 severely affected CoV replication [113]. Interestingly, MHV replicative structures are decorated with LC3 [106], generally regarded to as the non-functional precursor to the lipidated autophagosome marker LC3-II [122]. The involvement of autophagy and LC3 in DMV formation was further clarified when LC3 was shown to colocalize with MHV proteins nsp2/nsp3, dsRNA, and ER-associated degradation (ERAD) vesicle markers ER degradation-enhancing alpha-mannosidase-like 1 (EDEM1), Osteosarcoma amplified 9 (OS-9) in embryonic fibroblasts [113]. Down-regulation of LC3 inhibits MHV replication and virion production [113] whereas knocking out autophagy has no effect. Inhibition of MHV replication and virion production was attributed to a defect in DMV biogenesis, which negatively impacts non-structural protein production. These results suggest that LC3 and the ERAD pathway are necessary for DMV formation and the biogenesis of RTCs required for a productive infection. Finally, quantitative proteomics analysis revealed that SARS-CoV infection significantly upregulates BCL2-associated athanogene 3 (BAG3), a protein linked to regulation of the autophagy pathway [123]. Inhibition of BAG3 expression by RNA interference results in significantly reduced replication of SARS-CoV.

Unfortunately, conflicting data make it difficult to derive a definitive conclusion regarding the role of autophagy in CoV RTC formation. Some of these differences may be the result of using different cell lines and different CoVs. What is clear though is that proteins with known roles in autophagy are involved in CoV replication; however, the process of autophagy *per se* may not be functionally relevant to the formation of CoV DMVs.

### 4.3. The Secretory Pathway and CoV Replication

A number of studies indicate that the ER is involved in the biogenesis of the SARS-Co-V-induced reticulovesicular network (RVN), a membrane compartment involved in virus replication. Specifically, partial co-localization of CoV replicase proteins with the ER resident protein disulfide isomerase (PDI) [107] and the observation that the ER translocon subunit Sec61α redistributes to replicative structures during SARS-CoV infection support this idea [110]. In addition EDEMosome cargo proteins EDEM1 and OS-9, two proteins involved in ER quality control and ER associated degradation (ERAD), associate with CoV replicative structures [113]. However, many protein trafficking and membrane fusion proteins that function downstream of the ER in the early secretory pathway such as Sec13, syntaxin 5, GBF1, and Arf1 have not been detected at the RVN [110]. The involvement of the COPI complex was investigated using the drug BFA, which as mentioned above, blocks COPI-mediated vesicular transport at the ER-Golgi interface. When added to cells early in infection, BFA inhibits RVN formation and decreases, but does not completely abolish viral RNA synthesis [110]. Although the precise role of COPI is unknown, other positive RNA viruses discussed above such as PV and *Drosophila* C virus require the COPI-mediated vesicular transport for replication. This supports the notion of COPI as a common host factor required in viral infection.

## 5. *Togaviridae*

Togavirus virions are enveloped, spherical particles (50–70 nm in diameter) that contain a single-strand +RNA with a 5-cap and 3′ poly A-tail [124]. The family includes the genera *Rubivirus* and *Alphavirus.* Rubella virus (RUBV) is the sole member in the *Rubivirus* genus and is the causative agent of Rubella (also known as German Measles). The *Alphavirus* genus contains at least 30 members that are separated into New World and Old World viruses. The New World viruses include Venezuelan equine encephalitis (VEEV), Western equine encephalitis (WEEV) and Eastern equine encephalitis virus (EEEV). Old World alphaviruses evolved separately [125] and members include Semliki Forest virus (SFV), Sindbis virus (SINV), Chikungunya virus (CHIKV) and O’nyong’nyong virus. Transmission occurs mainly through mosquito vectors and human infections are often associated with fever, rash, severe joint pain (arthralgia) and stiffness that can last weeks to months in duration. Some pathogens in this group can cause much more severe illnesses including encephalitis in humans and animals.

Togavirus replication complexes originate from late endosomes and lysosomes and are morphologically similar for RUBV [126,127] and alphavirus-infected cells [128]. Alphavirus VRC biogenesis is comparatively well characterized and as such, we will focus on these structures. Most host factors that interact with alphavirus replication complexes were identified through pull down assays with alphavirus non-structural proteins. In this section we focused on host factors partners that have been speculated or clearly demonstrated to play functional roles in RC biogenesis or function. For a list of additional interacting partners that are not discussed here, readers are referred to the following articles: [129,130,131].

### 5.1. Alphavirus Replication

Shortly after alphaviruses infect host cells, small single-membrane bulb-shaped invaginations (~50 nm diameter) called “spherules” form on the external surface of the plasma membrane [132]. The spherules, which are associated with viral nonstructural proteins (nsPs) and dsRNA, each contain a neck-like opening to the cytoplasm (5–10 nm in diameter) that permits exchange of metabolites and export of nascent viral RNA [128]. The fact that dsRNA can be detected inside the spherules and the presence of partially processed non-structural proteins (P123 and nsP4) on the spherule necks [132], suggests that these structures are the sites of viral RNA synthesis [116]. Internalization of spherules by endo-lysosomal membranes gives rise to type 1 cytopathic vacuoles (CPV1), which are 600–2000 nm in diameter [24] (Figure 1C). The endosomal origin of CPVs is confirmed by the observation that these structures are often positive for both endosomal and lysosomal markers [128]. As infection progresses, the non-structural polyprotein precursors are further processed to yield individual non-structural proteins and negative-strand synthesis is inactivated. The fully processed non-structural proteins together form the mature replicase [115,133,134], which is required for efficient synthesis of positive-sense genomic and subgenomic RNA. Spherules are devoid of ribosomes and virus capsid proteins but these structures/proteins are frequently found juxtaposed to the spherule openings [128], suggesting that the site of translation is in close proximity to replication sites.

### 5.2. Membrane Lipids

The nsP1 protein of alphaviruses, which is required for 5’ capping of viral RNAs [135], is involved in attachment of the replication complex to membranes [136] via a highly conserved amphipathic helix [137]. When expressed in the absence of other viral proteins, nsP1 is targeted to the inner surface of the plasma membrane but this not sufficient for cytoplasmic vacuole formation [138]. NsP1 is modified by acylation; however, the significance of this process in its function has yet to be determined [139]. Cholesterol and sphingomyelin in the plasma membrane are important for alphavirus fusion [140,141,142] and budding [143,144]. Importance of the latter is evidenced by the observation that SINV infection of Niemann-Pick disease-A fibroblasts (NPAF), which cannot degrade sphingomyelin, results in reduced levels of genomic RNA as well as an altered ratio of subgenomic-to-genomic RNA. The authors suggest that due to the build-up of cholesterol and sphingolipids in late endosomes/lysosomes, biogenesis of replication complexes are negatively affected [114]. Interestingly, the alphavirus virions produced in NPAFs were 26 times more infectious than those produced in normal human fibroblasts; resulting in increased titers and cell death. This suggests that cellular production of less infectious virus may be a consequence of host restriction on virus replication.

### 5.3. Membrane Trafficking Proteins

The study of how alphaviruses enter and exit mammalian cells led to a number of fundamental discoveries about membrane trafficking. Therefore, it is somewhat surprising that comparatively little is known about how membrane trafficking components affect replication. However, it does appear that cytoskeletal elements are involved. Infection of cells with a recombinant SINV encoding GFP-tagged nsP3, followed by anti-GFP pull-downs revealed that this viral protein associates with cytoskeletal proteins, chaperones, elongation factor 1A, and heterogeneous nuclear ribonucleoproteins [115]. Others reported that nsP3 also binds actin, tubulin and myosin [145][144], vimentin (an intermediate filament protein) [115], and the cytosolic molecular chaperone Heat shock cognate protein 70 (Hsc70) [115,146]. These interaction data suggest that the SINV RCs associate with the cytoskeleton. The concern that the highly abundant cytoskeletal proteins were mere contaminants in the nsP3-binding studies [130] was partially assuaged by imaging studies showing that nsP3 associates with vimentin patches [115]. Imaging studies also provided evidence for Hsc70-ns3p having a role in alphavirus RC formation and/or function [115,146]. Some of the many functions of Hsc70 (reviewed in [147]) are to target proteins for degradation, regulate the translocation of proteins into different cellular organelles such as ER and mitochondria, and regulate apoptosis. Hsc70 has been linked to the replication of many viruses [148] but whether or not this is largely a reflection of its general role as a chaperone has yet to be determined.

NsP3 proteins of SFV, SINV, and CHIKV have also been shown to interact in an SH3 domain-dependent manner with amphiphysin-1 and Bin1/amphiphysin-2, both of which are involved in endocytosis and membrane trafficking [117]. The re-localization of amphiphysins to alphavirus RCs promotes replication, further solidifying the role of these host proteins in formation or stabilization of the replication sites. Finally, recent data suggest a role for phosphatidylinositol 3-kinases (PI3Ks) in alphavirus RC formation. Specifically, the activity of these kinases is required for the initial internalization of spherules at the plasma membrane as well as their subsequent trafficking on microtubule and actin networks [116].

### 5.4. Ras GTPase-Activating Protein-Binding Proteins

The RNA-binding proteins Ras GTPase-activating protein-binding protein (G3BP)1 and G3BP2 are structural components of stress granules: large cytoplasmic ribonucleoprotein complexes that function in regulating translation. Several studies have identified interactions between G3BP proteins and alphavirus nsP2 and nsP3 [115,129,130,146]. To differentiate between host proteins associated with replication complexes from those that interact with individual nsP2 or nsP3 proteins, Varjak *et al.* used small dextran-covered magnetic beads that incorporated into CPV-1 structures in infected cells, thus permitting the isolation of membranous vesicles. This study confirmed the enrichment of G3BP1 and G3BP2 in CPV-1 vesicles in SFV infected cells [131], however, it was not possible to determine when these host proteins were recruited. Interaction of G3BPs with alphavirus nsPs as evidenced by the observation that the insect G3BP1 homolog Rasputin, was detected in nsP3-containing complexes isolated from mosquito cells infected with SINV [146]. While G3BP proteins may serve an important and conserved function in alphavirus infections, it remains unclear as to how these host proteins function in RC biogenesis. While recruitment of G3PB proteins to RCs is a common feature of alphavirus infection, this was not observed in cells infected with the flavivirus YFV, indicating that G3BP is not a host factor for RCs of all positive strand RNA viruses [130].

SINV may recruit G3BP as a means to block G3BP-dependent export of host mRNAs to the cytoplasm or that host RNAs undergoing nuclear export are sequestered, resulting in translational shutoff. Alternatively, this may reflect a specific host response to counteract infection [130]. The interaction between nsP3 and G3BP1/G3BP2 in CHIKV-infected cells occurs late in the replication cycle where the bulk of G3BP1 and G3BP2 is not associated with the viral RC but rather, is sequestered in nsP3-G3BP aggregates in the cytoplasm [149]. This may indicate that G3BPs play different roles early and late in infection. For example, late in infection interaction between nsP3 and G3BPs, which are nucleating factors for stress granules, could prevent the formation of these RNA granules. Stress granules have been implicated in the antiviral response [150] and have been reported to form late during alphavirus infection, a process that correlates with host translational shutdown [151]. These seemingly contrasting observations may be explained by the temporal dynamics of the nsp-G3BP association (see above for CHIKV). Given the role of G3BPs in stress granule formation, it seems likely that nsPs associate with G3BPs as a means to inhibit their formation; a theory that is supported by recent data showing that translational shut-off in cells infected with the alphavirus VEEV (whose replication mechanisms do not appear to involve G3BPs) is comparatively slower [146].

## 6. Summary

A common feature of all +RNA viruses is their ability to modulate cellular membranes aiding in the concealment of replication product intermediates from recognition by cellular immune sensors. Despite differences in membrane curvature (DMVs, InVs, *etc*.), all replication complexes are made up of viral proteins, RNA, and cellular factors. In this review, we have focused on identifying host factors that are proposed or validated to play a role in the replication complexes of human pathogens belonging to four different virus families. The cellular proteins at the replication complexes include proteins involved in lipid metabolism, intracellular trafficking, autophagy, secretory pathways, transcription, and translation. Understanding precisely how these host proteins function in virus replication may open avenues for development of novel anti-viral therapeutics.

## Abbreviations

**Table A1:** Commonly used abbreviations in this review article.

Abbreviation	Full Nomenclature
ACACA	Acetyl-CoA carboxylase 1
ACBD3	Acyl-coenzyme A binding domain containing 3 protein
Arf	ADP-Ribosylation factor
ATG5	Autophagy protein 5
BAG3	Bcl-2-associated athanogene 3
BFA	Brefeldin A
ACACA	Acetyl-CoA carboxylase 1
ACBD3	Acyl-coenzyme A binding domain containing 3 protein
Arf	ADP-Ribosylation factor
ATG5	Autophagy protein 5
BIG	Brefeldin A-inhibited guanine nucleotide-exchange factor
CHIKV	Chikungunya virus
CM	Convoluted Membranes
CME	Clathrin-mediate endocytosis
CPV-1	Type 1 cytopathic vacuoles
CV	Coxsackie virus
DENV	Dengue virus
DMV	Double-Membrane Vesicles
dsRNA	Double-stranded RNA
EDEM1	ER degradation-enhancing alpha-mannosidase-like 1
EEEV	Eastern equine encephalitis virus
EV	Enterovirus
FAS	Fatty acid synthase
G3BP	Ras GTPase-activating protein (SH3 domain) binding protein
GBF1	Golgi brefeldin A resistant guanine nucleotide exchange factor 1
HCV	Hepatitis C virus
HMGCR	3-hydroxy-methyglutaryl-CoA reductase
HRV	Human Rhinovirus
Hsc70 (also known as HSPA8)	Heat shock cognate 71 kDa protein
JEV	Japanese Encephalitis virus
LAMP-1	Lysosomal-associated membrane protein 1
LC3 (Cytosolic form; LC-I)	Microtubule-associated protein 1A/1B-light chain 3
LC3-II (Lipidated form)	LC3-phosphatidylethanolamine conjugate
LDLR	Low density lipoprotein receptor
MERS-CoV	Middle East respiratory syndrome coronavirus
MHV	Mouse Hepatitis virus
MVD	Mevalonate diphosphate decarboxylase
NPAF	Niemann-Pick disease-A fibroblasts
OS-9	Osteosarcoma amplified 9, ER lectin
PC	Paracrystalline Array
PDI	Protein disulfide isomerase
PI	Phosphatidylinositol
PI4K	Phosphatidylinositol-4-OH kinase
PI4P	Phosphatidylinositol 4-phosphate
PtdCho	Phosphatidylcholine
PV	Poliovirus
RTC	Replication/Transcription Complex
RUBV	Rubella virus
RVN	Reticulovesicular Network
SARS-CoV	Severe Acute respiratory syndrome coronavirus
Sec-13, -31, -61	ER translocon proteins
SFV	Semliki Forest virus
SINV	Sindbis virus
SLEV	St. Louis Encephalitis virus
SREBP	Sterol-regulatory element binding protein
STMN1	Stathmin 1/oncoprotein 18
TBEV	Tick-borne Encephalitis virus
VEEV	Venezuelan equine encephalitis virus
VP	Vesicular Packet
VRC	Virus Replication Complex
WEEV	Western equine encephalitis virus
WNV	West Nile virus
YFV	Yellow Fever virus
